# Evaluating biometric indices for Indian Gagata, *Gagata cenia* (Hamilton, 1822) through multi-model inferences

**DOI:** 10.1016/j.heliyon.2022.e12739

**Published:** 2022-12-30

**Authors:** Zubyda Mushtari Nadia, Newton Saha, Prosun Roy, Md. Asif Iqbal, Most. Shakila Sarmin, Md. Yeamin Hossain

**Affiliations:** a Department of Aquatic Animal Health Management, Sher-e-Bangla Agricultural University, Dhaka, 1207, Bangladesh; b Department of Aquaculture, Bangladesh Agricultural University, Mymensingh, 2202, Bangladesh; c Department of Fisheries Management, Patuakhali Science and Technology University, Dumki, Patuakhali, 8602, Bangladesh; d Faculty of Fisheries, Patuakhali Science and Technology University, Dumki, Patuakhali, 8602, Bangladesh; e Department of Fisheries, University of Rajshahi, Rajshahi, 6205, Bangladesh

**Keywords:** *Gagata cenia*, Growth parameters, Size at sexual maturity, Optimum catchable length, Mortality, Jamuna River

## Abstract

Biometric indices provide crucial information on fish population growth and aquatic habitat well-being. This study offers the first comprehensive elucidation on biometric indices of *Gagata cenia* (Hamilton, 1822) specifically; population structure, growth pattern and parameters, form factor (*a*_*3.0*_), conditions factors, prey-predator status, reproduction, optimum catchable length (*L*_*opt*_), mortality and exploitation rate in the Jamuna River, northern Bangladesh. The aim of this study is to estimate stock status of *G. cenia* in order to protect the declining wild stock. Total of 725 fishes were randomly collected (ranges between 2.7 and 8.5 cm total length (TL), while 0.30–8.24 g body weight (BW)) occasionally using conventional fishing gears during June 2020 to May 2021. The *b* value of length-weight relationship (TL *vs.* BW) was 3.33 which indicated positive allometric growth pattern with *r*^*2*^ value 0.942. Growth parameters were supposed as asymptotic length (*L*_*∞*_) = 9.09 cm, (*K*) = 0.94 year^−1^, growth performance index (*Ø′*) = 1.89 and longevity (*t*_*max*_) = 3.2 year^−1^ for this population. The calculated *a*_*3.0*_ was 0.0172 and Fulton’s condition (*K*_*F*_) is the best suited tool for assessing the well-being of this population. Additionally, relative weight (*W*_*R*_) specifies an imbalanced territory with regard to the occurrence of predators. The size at sexual maturity (*L*_*m*_) and *L*_*opt*_ for combined sexes of *G. cenia* were 5.4 cm and 6.0 cm, respectively henceforth fish sizes 6.0–6.5 cm TL are recommended for exploitation. The current study verified fishing mortality was significantly lower than natural mortality and the exploitation rate (*E*) was 0.32 which indicated the stock was under fishing. Our findings would be very beneficial in planning the sustainable and appropriate management of this fish in Bangladesh and nearby ecosystems.

## 1 Introduction

Fishes of Siluriformes are commonly known as catfish, one of the largest orders of teleosts containing more than 4000 species representing near 12% of all teleosts [1,2]. Catfish are highly diverse and distributed worldwide. The Indian Gagata, *Gagata cenia* [3], is a freshwater catfish belonging to the family Sisoridae [4]. In India, Bangladesh, Pakistan, Myanmar, and Nepal, this fish is available in the Indus, Mahanadi, Ganges, Irrawaddy, Salween, and Brahmaputra drainages [[4], [5], [6]]. In *G. cenia*, the dorsal fin spine is serrated on both edges, pectoral fin without filamentous prolongation and three bands on head and its dorsal with dark saddles extending ventrally only to the lateral line and caudal fin with a transverse black bar across peduncle and round or square black spot on the middle of each lobe [7,8].

For being smaller in size (less than 25 cm) it is categorized as a small indigenous fish species in Bangladesh which is locally known as Benia, Jungla, Kauwa, Gang tengra, Gang magur [6,9]. The amount of catch of this fish is not much in water bodies of Bangladesh, but mostly found in the Padma River, the Someswari River, the Mohanonda River, the Brahmaputra River, Chalan Beel, the Chhoto Jamuna River, the Atrai River, the Payra River and Tanguar Haor [6]. *G. cenia* is a commercially important fish species and is used for human consumption in Bangladesh [4]. It is a demandable fish for the people of Bangladesh with lower income as an important source of animal protein. Moreover, being a small indigenous fish species, *G. cenia* is a good source of vitamins and minerals that helps to eliminate malnutrition, mostly for poor women and children in Bangladesh [10,11]. However, this species has declined from its natural habitat. It is grouped into the least concern category in the aspect of both Bangladesh and the world [9,12]. Wild *G. cenia* is under threat from a variety of environmental factors, including habitat destruction, drying out of water sources like rivers, habitat changes and indiscriminate fishing [13,14]. Moreover, freshwater fishes are a vulnerable group of aquatic species due to their sensitivity to the alteration of the aquatic environment and habitat [15]. *G. cenia* is not paid much attention by the researchers of Bangladesh and other countries of the world. For this purpose, research work is needed for collecting necessary information about this fish and its present status for better management to gain sustainable production.

Biometric indices of a fish species from a specific water body reflect the condition of fish, the ecosystem and suitability of the water body for their survival [16–18]. The traits include growth pattern, the number of offspring, size and sex ratio of offspring, the seasonal variation of reproduction, age and dimension of matured fish groups and growth type, and natural mortality whereas all the factors are interconnected [19]. The most significant aspect of biometric evaluation is determining length-weight relationships (*LWRs*), i.e., fish growth parameters. In fisheries, length-weight relationships (*LWRs*) are highly useful for projecting length distributions into weights for biomass assessment [20]. Fish size classes may influence the distribution of individuals within a fish population [21]. For generating ecosystem models, knowledge of growth parameters such as the Von Bertalanffy growth coefficient (*K*), asymptotic length (*L*_*∞*_), age at zero length (*t*_*0*_), maximum reported age (*t*_*max*_), size at sexual maturity (*L*_*m*_) and natural mortality (*M*) is required [22]. The form factor (*a*_*3.0*_) is a measure of the body shape of each fish in a stock [23]. Moreover, the size at sexual maturity (*L*_*m*_) is a key management metric for determining if enough juveniles in a collected stock are mature or ready to reproduce [24]. Besides, morphometric relationships such as length-weight relationships (*LWRs*), relative condition factor (*K*_*R*_), and Fulton’s condition factor (*K*_*F*_) are essential biological metrics for fishes that may be used to determine the state of stocks and the health of fish populations [25].

A necessary precursor to the advancement of trait-based studies in fish ecology is the advancement and exploration of large databases containing eco-biological characteristics of species representing a diverse taxonomic group [26–28]. The river-based ecosystem is influenced by both natural and anthropogenic indicators and for survival in such an ecosystem fish must undergo a continuous physiological change as a part of adaptation [16,27,29]. For many years, riverine ecosystems have been suffering from intense human intervention resulting in habitat loss and degradation and as a consequence, many fish species have become highly endangered, particularly in rivers where heavy demand is placed on freshwaters [17]. *G. cenia* is well distributed and found in the Jamuna River which is one of the longest rivers and it is believed to be an important spawning and feeding ground for riverine fish species of Bangladesh [30,31].

Only few studies have been done on this species from different water bodies such as length-weight and length-length relationships by Islam and Mia [32] from the Atrai River, Bangladesh; [33] from the Payra River, Bangladesh; Nahar et al. [34] from the Ravi River, India; condition factor Sharma et al. [35] from the Padma River, Bangladesh; and parameters associated with growth factors [36] from the Atrai River. Nonetheless, a study on *G. cenia* from the Jamuna River in detail [3] has not been carried out yet. Detailed information on their life-history traits is required for proper management of this species and the implementation of conservation policies for the Jamuna River [37,38]. The knowledge of biometric indices or population biology is indispensable to protecting the wild stock of this valuable species. Accordingly, to evaluate the status of *G. cenia* stock and for their better management*,* this study focuses on describing the biometric indices including length frequency distribution (*LFD*), length-weight relationship (*LWR*), growth parameters (asymptotic length, asymptotic weight, growth coefficient, age at zero length), growth performance index, life-span, condition factors, relative weight (*W*_*R*_), form factor (*a*_*3.0*_), reproduction (size at sexual maturity, *L*_*m*_; age at maturity, *t*_*m*_), mortality and exploitation rate from the Jamuna River in Bangladesh.

## 2 Materials and methods

### 2.1 Study region and sampling

**Fig. 1:**
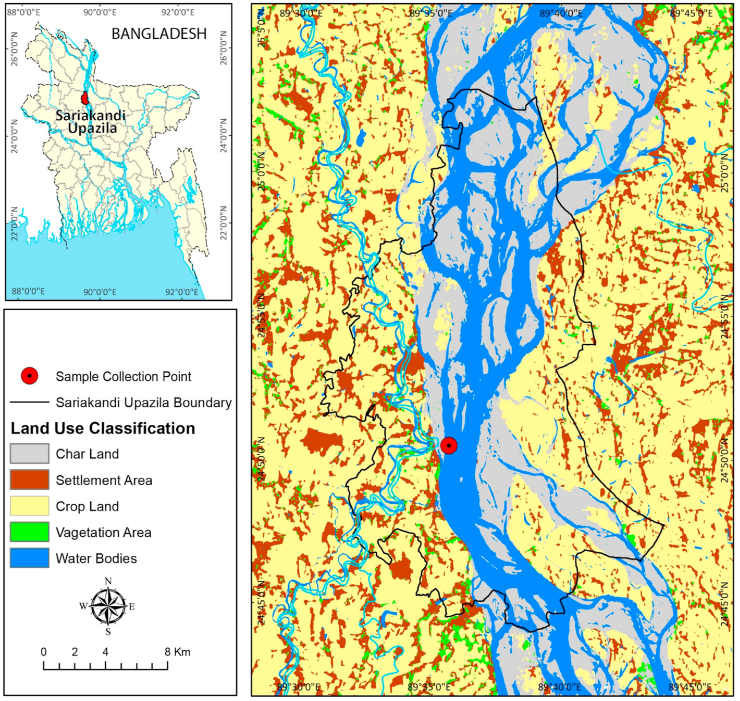
Map showing location of the study area (red mark circle indicates sampling point) for *G. cenia* [3] in the Jamuna River, northern Bangladesh. (For interpretation of the references to colour in this figure legend, the reader is referred to the Web version of this article.)

The Jamuna River is one of the major rivers of Bangladesh which is the main distributary of the Ganges, and the river is a diversity rich water body [39]. From the artisanal fisher’s catch of Jamuna River (Sariakandi Upazila point; Lat. 24°51′12″N and Long. 89°36′19″E), a total of 725 individuals of *G. cenia* were collected over a period during June 2020 to May 2021 (Fig. 1 [3]), through different conventional fishing tools namely-cast net containing mesh size of 1.0–2.0 cm, lift net having mesh size of less than 0.5 cm and gill net containing mesh size of 1.5–2.5 cm. After sampling, samples were stored immediately in an icebox and then transferred into the 10% buffered formalin solution in the laboratory for fixation of the samples. For the measurement of total length (TL) and standard length (SL) of each specimen wooden measuring board was used to the 0.01 cm accuracy. Moreover, a digital balance machine (AND, FGH-400, Korea) was used to measure body weight (BW) to 0.01 g accuracy.

### 2.2 Population structure

The length frequency distribution is used to reveal the age, growth and population structure of the target fish species. Length frequency distributions (*LFDs*) for the population of *G. cenia* were revealed through 0.5 cm class intervals of total length (TL). The length composition of a fish population frequently displays modes among fishes with short spawning seasons and uniform development which can be considered for determining the modal length of the first age groups.

### 2.3 Growth pattern

Among the growth parameters of biometric indices, length-weight relationship of fish is the most important and most widely used. Evidence showed that for the management of open water fish diversity *LWR* estimation is a convenient way if the data of length-weight of the sample is available [40,41]. Moreover, standing crops as well as seasonal fluctuations of fish growth can be calculated using the length-weight relationship.

The following formula was used to evaluate the relationship between length and weight and the equation is *W* = *a* × *L*^*b*^; where, W is the total weight (g), L is the total length (cm), and the regression parameters are *a* and *b*. The *a* and *b* of the length-weight relationship are calculated by linear regression analysis from the natural logarithms: ln (*W*) = ln (*a*) + *b* ln (*L*) [29,42]. The highest deviation was avoided from the analyses based on the statement of Froese [42]. Therefore, *t*-test was applied for determining the significant deviation from the isometric value (for length-weight relationship, *b* = 3 and for length-length relationship, *b* = 1) [43]. The deviation of b from the hypothetical isometric value indicates that allometric growth can be either positive (*b* > isometric value) or negative (*b* < isometric value). Analysis of covariance (ANCOVA) [44] was applied for assessing significant dissimilarities in slopes and intercepts among the relationships. Moreover, the *LLR* (TL-SL) was analyzed using a linear regression model [45].

### 2.4 Growth parameters

To explain growth parameters (*L*_*t*_, mean length at age *t*; *t*, the age year^−1^, and *t*_*0*_, the hypothetical age of zero length), the Von Bertalanffy (VBG) model was used, with *L*_*t*_ = *L*_*∞*_ [1 − exp {−*K* (*t* − *t*_*0*_)}] for length basis [46]. Furthermore, the growth parameter, asymptotic length (*L*_*∞*_) was estimated based on the highest length recorded using the following formula: log (*L*_*∞*_) = 0.044 + 0.9841 × log (*L*_*max*_) [47]. The age at zero length was studied with the calculation of log (−*t*_*0*_) = - 0.3922–0.2752 log *L*_*∞*_ − 1.038 log *K* [48] and the other parameter, growth coefficient (*K*) was assessed through the formula of *K* = ln (1 + *L*_*m*_/*L*_*∞*_) *t*_*m*_ [49]. Moreover, the asymptotic weight (*W*_*∞*_) was calculated through *W*_*∞*_ = *a* × *L*_*∞*_^*b*^ and the growth performance index was evaluated using the formula of *Ø'* = log10*K* + 2 log10*L*_*∞*_ [50]. Using the following formula of *t*_max_ = 3/*K*, lifespan or longevity was calculated [50].

### 2.5 Form factor (a_3.0_)

*G. cenia* form factor (*a*_*3.0*_) was calculated using Froese’s [42] formula: *a*_*3.0*_ = 10^log^·^*a*-s (*b*−3)^, where *a* and *b* are *LWR* (TL vs. BW) regression variables, and s is the slope of log a vs. b. We used a mean slope S = −1.358 since there was no detail on *LWR* for this species [42] to analyze the form factor and calculate the regression (S) of log *a vs. b*.

### 2.6 Condition factors

The condition factor reveals the well-being of fish [51]. Tesch’s [52] equation was used to determine the allometric condition factor (*K*_*A*_): *K*_*A*_ = *W*/*L*^*b*^, where *W* is the BW in g, L is the TL in cm, and b is the *LWRs* parameter. The condition factor (*K*_*F*_) was calculated using Fulton’s formula [53]: *K*_*F*_ = 100 (*W*/*L*^*3*^) (where *W* is the body weight in g and L is the TL in cm). A scaling factor of 100 was used to get the *K*_*F*_ close to the unit [42].

Moreover, the relative condition factor (*K*_*R*_) was evaluated through the following formula of Le Cren [54]: *K*_*R*_ = *W/(a* × *L*^*b*^*)*, where *W* is the body weight (g), *L* is the total length (cm), and *a* and *b* are the *LWR* parameters. The following equation was used to assess relative weight (*W*_*R*_): *W*_*R*_ = (*W*/*W*_*s*_) × 100 Froese [42]; where W is the weight of a single species and *W*_*s*_ is the expected normal weight as intended by *Ws* = *a* × *L*^*b*^ (here, the values of *a* and *b* are calculated from the equation of TL vs BW).

### 2.7 Size at sexual maturity (*L*_*m*_) and optimum catchable length (*L*_*opt*_)

The empirical formula of Binohlan and Froese [55] was used for the assessment of size at sexual maturity (*L*_*m*_) of *G. cenia* and the formula is as follows: *log* (*L*_*m*_) = −0.1189 + 0.9157 × *log* (*L*_*max*_); where the *L*_*m*_ = size at sexual maturity in TL; *L*_*max*_ = maximum observed length (TL) of *G. cenia* in the present study. In addition, the age at maturity (*t*_*m*_) was evaluated through the equation of *t*_*m*_ (50%) = (−1/1) × ln (1 − *L*_*m*_/*L*_*∞*_) [56].

Additionally, *L*_*opt*_ was assessed by the model of Beverton [49]: *L*_*opt*_ = *L*_*∞*_ [3/(3 + *M*/*K*)]; where, *L*_*∞*_ = asymptotic length as projected by log (*L*_*∞*_) = 0.044 + 0.9841 × log (*L*_*max*_) [47]; *M* = natural mortality and *K* = growth coefficient *K* = 3/*t*_*max*_ [50]. For the management of stock, we applied Froese’s [57] indicator (i) percentage of the mature individual in the catch, with 100% as the target; (ii) ± 10% of optimum length from the frequency of fish, with 100% as a target; and (iii) plus 10% *L*_*opt*_ of mega-spawners from stock structure, with 0% as the target.

### 2.8 Mortality and exploitation rate

From length-frequency distribution data and growth factors, the parameters of mortality (*Z*, *M*, and *F*) were calculated. The instantaneous total mortality (*Z*) was evaluated using the following formula: ln (Nt/Δt) = a + b × *t* (*N*_*t*_ is an individual number of relative age (*t*) and Δt, the time required) [50]. Besides, the natural mortality was acquired by *M* = −ln [0.01]/*t*_*max*_ [56]. Fishing mortality was estimated with *F* = *Z* – *M*. The formula *E* = *F*/(*F* + *M*) was used to calculate the rate of exploitation [58].

### 2.9 Statistical analysis

Microsoft® Excel-add-in DDXL and GraphPad Prism 8 software were applied for data processing. In case of evaluating the association of condition factors with total length (TL), the Spearman rank-correlation test was applied. Whereas, Wilcoxon sign-ranked test was used to link the average relative weight (*W*_*R*_) with 100 [59]. Moreover, all the statistical analyses were performed at 5% (*p* < 0.05) level of significance.

## 2.10 Ethical statement

After satisfying their standards, the Ethics Committee of Patuakhali Science and Technology University (PSTU) authorized the experiment’s design and execution.

## 3 Results

### 3.1 Population structure

**Fig. 2:**
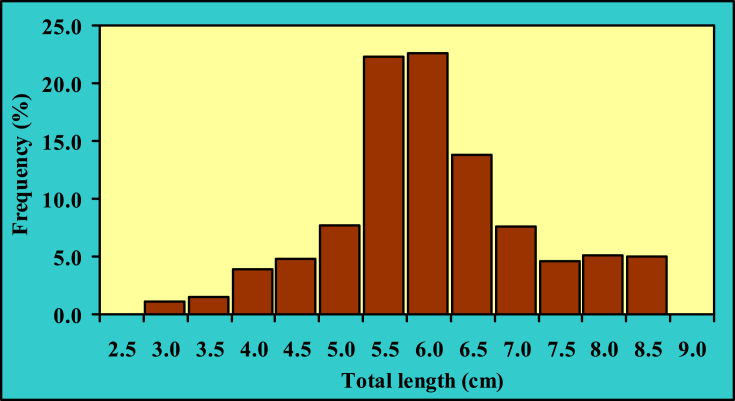
Length-frequency distribution of *G. cenia* [3] from the Jamuna River, northern Bangladesh.

Explanatory statistics on length (cm) and weight (g) measurements with their 95% confidence interval (CI) are represented in Table 1. The *LFDs* of *Gagata cenia* point out that the smallest and largest individuals were 2.7 and 8.5 cm TL, respectively, whereas the BW varied from 0.30 to 8.24 g. The maximum population stands on 5.5–6.0 cm TL size group and constituted 44.97% of the total population in the Jamuna River (Fig. 2 [3]). Based on the Shapiro-Wilk normality test, the *LFDs* were not normally distributed (*p* < 0.0001 for TL and *p* < 0.0001 for BW) for the population of *G. cenia* in Jamuna River (Northern Bangladesh).

**Table 1:** Explanatory statistics on length (cm) and weight (g) measurements with their 95% confidence interval of *G. cenia* in the Jamuna River.

Measurement	*n*	Minimum	Maximum	Mean ± SD	CI_95%_
Total length (cm)	725	2.7	8.5	5.84 ± 1.14	5.75–5.92
Standard length (cm)		1.9	7.2	4.75 ± 0.95	4.68–4.82
Body weight (g)		0.30	8.24	2.54 ± 1.74	2.42–2.67

Here, *n*, sample size; CI, Confidence interval for mean values.

### 3.2 Growth pattern

In Table 2; the regression parameter, 95% CL of *a* and *b*, and the coefficient of determination (*r*^*2*^) and growth pattern (GP) of *G. cenia* are specified. The calculated allometric coefficient (*b*) of TL vs. BW indicates positive allometric growth type in the combined sexes of *G. cenia* (*b* > 3.00; *p* < 0.0001) from the Jamuna River. Further, the *b* value of *LLR* (SL vs. TL) also indicates the positive allometric growth pattern (*b* > 1), as demonstrated in Table 2. Both relationships (LWR and LLR) were highly significant (*p* < 0.0001), with all *r*^*2*^ values being ≥0.992.

**Table 2:** Descriptive statistics of length-weight and length-length relationships of *G. cenia* in the Jamuna River.

Formula	*n*	Regression variables		*a* (±95% CI)	*b* (±95% CI)	*r*^*2*^	GP
		a	b				
BW = *a* × TL^*b*^	725	0.0061	3.33	0.0055 to 0.0068	3.27–3.39	0.942	A+
TL = *a* + *b* × SL		0.1284	1.20	0.0889 to 0.1678	1.19 to 1.21	0.992	A+

Here, *n*, Sample size; BW, Body weight; TL, Total length; SL, Standard length; *a*, Intercept; *b*, Slope; CI, Confidence interval for mean values; *r*^*2*^, Coefficient of determination, GP, growth pattern; and A+, Positive allometric.

### 3.3 Growth parameters

**Fig. 3:**
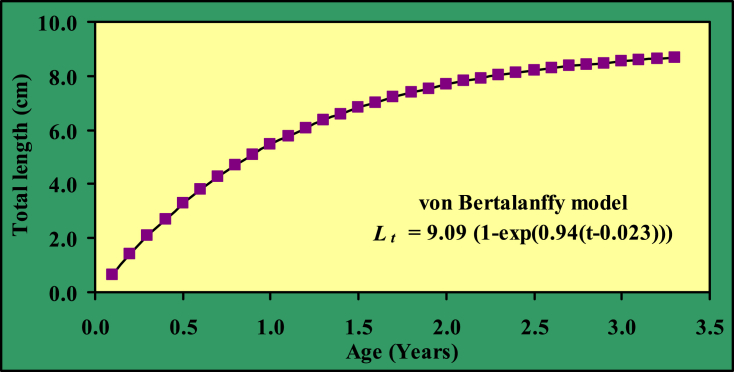
The Von Bertalanffy growth curve based on length of *G. cenia* [3] from the Jamuna River, Bangladesh.

The Von Bertalanffy growth parameters obtained for *G. cenia* were *L*_*∞*_ = 9.09 cm, *W*_*∞*_ = 9.49 g, *K* = 0.94 and *t*_*0*_ = 0.023 cm. The growth performance index (*Ø′*) was 1.89. The longevity of age *t*_*max*_ was 3.2 years (Table 3). The observed and predicted maximum length was 8.5 and 9.09 cm, respectively (Fig. 3 [3]).

**Table 3:** Growth, reproduction, and mortality parameters of *G. cenia* in the Jamuna River.

Parameters	Values
Asymptotic length in cm (*L*_*∞*_*)*	9.09 cm
Asymptotic weight in g (*W*_*∞*_*)*	9.49 g
Growth coefficient per year (*K)*	0.94
Age at zero length in cm (*t*_*0*_)	0.023 cm
Growth performance index (*Ø′*)	1.89
Life-span/Longevity in year (*T*_*max*_)	3.2 years
Size at sexual maturity in cm (*L*_*m*_)	5.4 cm
Age at maturity in year (*t*_*m*_)	0.90 year
Natural mortality per year (*M*)	1.44 year^−1^
Total mortality per year (*Z*)	2.12 year^−1^
Fishing mortality (*F*)	0.68 year-1
Exploitation per year (*E*)	0.32
Optimum catchable length (*L*_*opt*_)	6.06 cm

### 3.4 Condition factors and prey-predator status

**Fig. 4:**
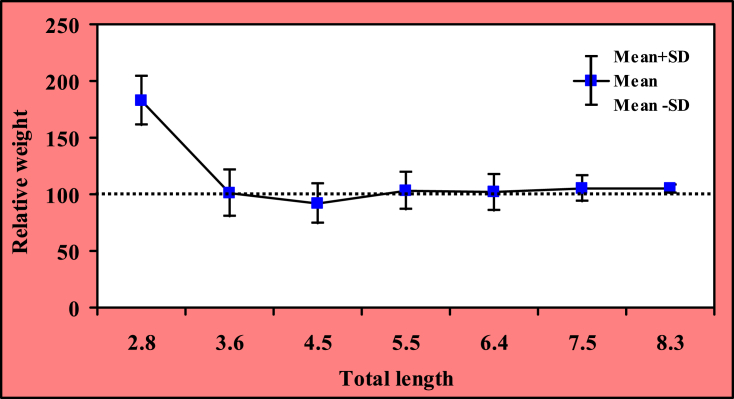
Relationship between total length and relative weight of *G. cenia* [3] in the Jamuna River.

All the condition factors including *K*_*A*_, *K*_*F*_, *K*_*R*_ and *W*_*R*_ with mean values and 95% CL are presented in Table 4. Additionally, the relationships of condition factors with TL are presented in Table 4. Results indicated that all the condition factors were significantly correlated with TL. Moreover, *W*_*R*_ demonstrated very significant differences from 100 (Wilcoxon signed rank test, *p* < 0.0001) indicating the imbalance population with the presence of predator and prey. In addition, the relative weight (*W*_*R*_) in relation to total length (TL) is shown in Fig. 4 [3].

**Table 4:** Descriptive statistics on condition factors measurements with their 95% CI; and estimation of correlation for condition factors with total length (TL, cm) of *G. cenia* in the Jamuna River.

Conditions	Minimum	Maximum	Mean ± SD	CI_95%_
*K*_*A*_	0.0042	0.0138	0.0071 ± 0.0012	0.0070 to 0.0072
*K*_*F*_	0.6261	1.7781	1.1008 ± 0.1945	1.0867 to 1.1151
*K*_*R*_	0.6207	2.1108	1.0219 ± 0.1770	1.0090 to 1.0348
*W*_*R*_	62.07	211.08	102.19 ± 17.71	100.90 to 103.48

Relationships	*r*_*s*_ values	CI_95%_ of *r*_*s*_	*p* values	Degree of significance
TL versus *K*_*A*_	0.2203	0.1477 to 0.2904	<0.0001	****
TL versus *K*_*F*_	0.4218	0.3582 to 0.4816	<0.0001	****
TL versus *K*_*R*_	0.1527	0.07868 to 0.2251	<0.0001	****
TL versus *W*_*R*_	0.1527	0.07868 to 0.2251	<0.0001	****

Here, Condition factors (*K*_*A*_, Allometric; *K*_*F*_, Fulton’s; *K*_*R*_, Relative; *W*_*R*_, Relative weight); CI, Confidence interval; *r*_*s*_, Coefficient of spearman rank correlation test values; *p*, Exhibitions the intensity of significance; and ****Extremely significant.

### 3.5 Form factor

The *a*_*3.0*_ was observed as 0.0172 for combined sexes of *G. cenia* in the Jamuna River and the estimated value signifies this fish is short and deep in body shape. Furthermore, using existing data, *a*_*3.0*_ of *G. cenia* obtained from different waters around the world was calculated and represented in Table 5 [32–36].

**Table 5:** Calculated asymptotic length, form factor, size at sexual maturity, age at maturity, natural mortality, and optimum catchable length of *G. cenia* obtained from different waterbodies around the world.

Water body	Sex	Regression parameter		*L*_*max*_ (cm)	*L*_*α*_	*a*_*3.0*_	*L*_*m*_ (cm)	*t*_*m*_ (y)	*M* (y^−1^)	*L*_*opt*_	References
		a	b								
The Padma River, Bangladesh	M	–	–	8.1	8.67	–	5.16	0.90	–	5.78	[35]
	F	–	–	9.0	9.62	–	5.69	0.89	–	6.41	
	C	–	–	9.0	9.62	–	5.69	0.89	–	6.41	
The Atrai River, Dinajpur, Bangladesh	C	0.0060	3.06	7.3	7.83	0.007	4.70	0.92	1.51	5.22	[32]
Payra River, Southern Bangladesh	C	0.0035	3.17	11.7	12.45	0.006	7.23	0.87	1.12	8.30	[33]
The Ravi River, North-western India	C	0.0036	3.15	13.4	14.23	0.006	8.19	0.86	1.02	9.49	[34]
The Atrai River, Dinajpur, Bangladesh	C	0.0060	3.06	7.3	7.83	0.008	4.70	0.91	1.51	5.22	[36]
Jamuna River, Northern Bangladesh	C	0.0061	3.33	8.5	9.09	0.017	5.40	0.90	1.63	6.06	Present study

Here, M, Male; F, Female; C, Combined; *a* and *b* are Length-weight relationship parameters; *L*_*max*_, Maximum length; *L*_*α*_, Asymptotic length; *a*_*3.0*_, Form factor; *L*_*m*_, Size at sexual maturity; *t*_*m*_, Age at maturity; *M*, Natural mortality; and *L*_*opt*_, Optimum catchable length.

### 3.6 Size at sexual maturity and optimum catchable length

**Fig. 5:**
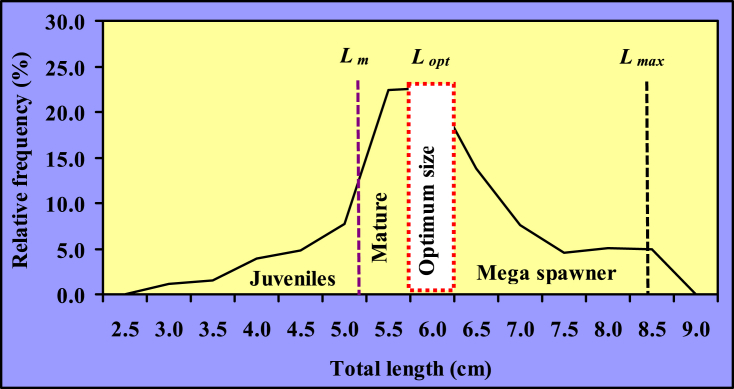
Optimum catchable length of *G. cenia* [3] in the Jamuna River (*L*_*m*_-size at sexual maturity, *L*_*opt*_*-*optimum catchable length, *L*_*max*_*-*maximum length).

The *L*_*m*_ for the population of *G. cenia* was estimated as 5.4 cm TL in the Jamuna River. Besides, the age at maturity (*t*_*m*_) was calculated as 0.90 year. The researchers conjointly calculated the *L*_*m*_ of *G. cenia* from numerous water bodies worldwide exploitation available knowledge to match the findings with the present study (Table 5). The estimated optimum catchable length (*L*_*opt*_) for *G. cenia* was 6.06 (∼6.0) cm TL in the Jamuna River, northern Bangladesh (Table 5 and Fig. 5 [3]).

### 3.7 Mortality and exploitation rate

**Fig. 6:**
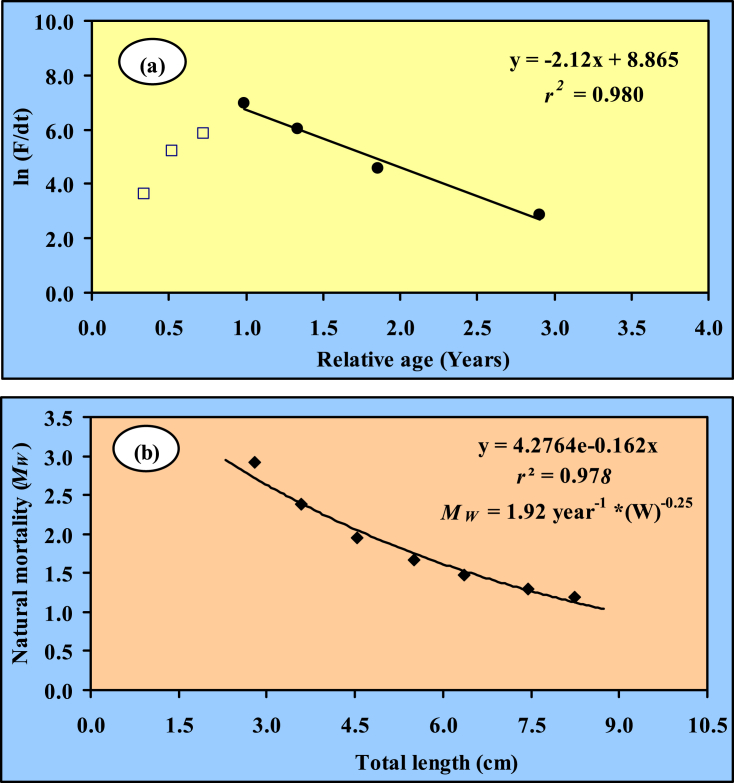
(a) Total mortality and (b) natural mortality of *G. cenia* (Hamilton, 1822) in the Jamuna River. Data included in the regression are shown as black solid points.

During the study, the total mortality assessed from the slope of the length converted catch curve was *Z* = 2.12 year^−1^ for *G. cenia* [Table 3 and Fig. 6(a)]. The calculated natural mortality (*M*) was 1.44 year^−1^ and fishing mortality (*F*) was 0.68 year^−1^. Moreover, the *M* value was very high for individuals when the species was less than 3.0 cm TL; on the contrary, it decreased with larger body sizes [Fig. 6(b)]. Moreover, the researchers used existing literature to calculate the *M* of *G. cenia* from waters around the world (Table 5). From the evaluated fishing and total mortality, the exploitation rate was calculated as *E* = 0.32 indicating under exploitation of this species.

## 4 Discussion

The present study was conducted in the Jamuna River, northern Bangladesh. We collected 725 samples with size ranges of 2.7–8.5 cm from the fisher’s catch during June 2020 to May 2021. Information on the population parameters of *G. cenia* is quite scant in literature from Bangladesh as well as worldwide. Hence, the current study intends to focus on the population parameters including growth patterns, condition factors, form factor, reproduction and mortality of *G. cenia* using empirical models [47,55] where the fish being studied couldn’t be collected for 12 consecutive months. Furthermore, this is the first time that empirical models have attempted to estimate population parameters of this species, so it can be used as baseline information for future detailed studies.

It was not feasible to sample *G. cenia* smaller than 2.7 cm in TL in this study, which might be owing to a lack of smaller individuals in the fishing grounds or the selectivity of fishing gears [18,[60], [61], [62], [63]] and additionally since smaller fish is thrown away by the fishers [64,65]. Besides, the maximum size of *G. cenia* was recorded as 8.5 cm TL which is lower than the maximum recorded value of 15 cm SL [4] and also recorded (11.7 cm TL) from Payra River, Bangladesh [33]; however higher than 7.3 cm TL by Islam and Azom [36]. The consistency could be due to the choice of fishing gear or simply because the fishers did not go to the spots with larger fish [18,66].

In this study, the estimated *b* value of the *LWR* (TL vs. BW) was 3.33 which was found to be accordant with the expected range (2.0–4.0) offered by Tesch [67] and (2.50–3.50) stated by Froese [42]. Parallel growth patterns were exposed in the species (*b* = 3.17) by Nahar et al. [33] from the Payra River and in the Ravi River (*b* = 3.15), north-western India [34]. On the contrary, *b* value was reported as 3.05 indicating isometric growth in the Atrai River, Dinajpur, Bangladesh [32] which is utterly unparalleled with our finding.

The growth parameters must be determined in order to forecast future yields and stock biomass from a certain aquatic environment [68]. We estimated the *L*_*∞*_ higher than our greatest specimen might be attributed to the Von Bertalanffy model being insufficient for assessing the growth of fish species as fish do not develop linearly [69]. At the juvenile stage, fish develop at a faster rate, whereas at the adult stage, fish grow at a slower rate [70]. As a result, the growth performance index (*Ø′*) is a more reliable technique for comparing the growth performance of a fish population since it incorporates both *L*_*∞*_ and *K* at the same time [50]. In this current study, the calculated growth parameters *L*_*∞*_, *Ø′* and *K* were 9.09 cm, 1.89 and 0.94 year^−1^, respectively for *G. cenia*. We also discovered the life span (*t*_*max*_) was 3.2 year and age at zero length (*t*_*0*_) was 0.023 cm. Attributable to the absence of related reference in regard to the growth parameters of this fish species, it was impractical to make comparisons across the water bodies.

Froese [42] suggested, the form factor assists to check whether or not the body form of individual fish in a particular population or else explicit species is considerably divergent from others. In this current study, the *a*_*3.0*_ value was 0.0172 while Islam and Azom [36] reported *a*_*3.0*_ value of 0.008 for this species from the Atrai River, Dinajpur, Bangladesh. The assessed a_3.0_ for the population of *G. cenia* was inside the cutoff points 0.0172–0.0193 narrated by Froese [42]; proposing short and deep body shape in the Jamuna River.

In this research, we have focused on four types of condition factors (*K*_*A*_, *K*_*F*_, *K*_*R*_, and *W*_*R*_) to assess the health and environmental status of *G. cenia* in the Jamuna River, nevertheless, most of the investigations address only a single condition feature. According to the Spearman rank correlation test, only Fulton’s condition factor showed the highest correlation values between TL vs. *K*_*F*_ (*r*_*s*_ = 0.4218) compared to other condition variables. That’s why, it’s reasonable to assume that *K*_*F*_ is the best for evaluating the well-being of this population in the Jamuna River, northern Bangladesh as well as the adjacent ecosystem.

In addition, based on the Wilcoxon-signed rank test, the mean *W*_*R*_ exposed eminently unique from 100 (*p* < 0.0001) connoting an imbalanced territory with food accessibility relative to the occurrence of predators [59,71] for *G. cenia* in the Jamuna River. As indicated by Rypel and Richter [72]; *W*_*R*_ can be used to judge the general physical status and wellness together with ecosystem disruptions at the population level. There is no literature attainable dealing with *W*_*R*_ of *G. cenia* prevents comparison with the present study.

This study revealed that the *L*_*m*_ and *t*_*m*_ for *G. cenia* was 5.4 cm and 0.90 year respectively in the Jamuna River and the *L*_*opt*_ was found to be 6.0 cm, suggesting the size range where the optimum yield might be acquired. From our study, 30% of mature fish were caught which were excluded from reproduction. The optimum size is slightly larger than the maturity size and the percentage was about 36. Furthermore, the mega-spawner group contributed about 22% of the caught stock (Fig. 5). To fulfill the catch of 100% mature fish, the first mature individuals remain to be unexploited before their spawn. To get healthier and more fecund fish with long-time surviving larvae mega-spawners must be protected. According to Froese [57]; prohibiting catching first mature fish and letting them grow up to optimum length of 6.0 cm and greater than 5.5 cm size fish as well as 6.0–6.5 cm size group of G. *cenia* are suggested for exploitation. Comparing this value with the calculated *L*_*m*_ of other water bodies, it’s found that the values are almost similar to the calculated value of the Atrai River, Dinajpur, Bangladesh [32,36] and Payra River, Bangladesh [33], but is much lower than the calculated value of the Ravi River, North-western India (Table 5), which may be attributed to the geographical variation. Tactlessly, this research conveys the first effort to estimate the size at sexual maturity and optimum catchable length for this fish, hence comparisons with other findings will be required in the future.

The mortality rate is important for determining the abundance of a fish species and setting harvest restrictions to maximize the value to the resource’s stakeholders. Natural mortality (*M*) was recorded as 1.44 year^−1^, while fishing mortality (*F*) was found as 0.68 year^−1^. Comparing this value with the calculated *M* of other water bodies, it is found that the value is almost similar to all the calculated values of worldwide waterbodies (Table 5). This study confirmed that fishing mortality was significantly lower than natural mortality which is also the first global estimate. One of the major causes of higher *M* was the predator in the Jamuna River which we revealed from the relative weight (*W*_*R*_) analysis through the Wilcoxon-signed rank test. The higher natural mortality may also be attributed to diseases, old age, predation, spawning stress and starvation which we did not consider in the present study. The lower value of natural mortality and estimated exploitation rate *E* (0.32) indicated under-fishing during that period. According to Gulland’s [73] assumption, the suitable yield is optimized when *F* = *M* (i.e., when *E* is more than 0.50, the stock is generally considered to be over-fished). The exploitation for *G. cenia* indicated that the stock was under-exploited, and the effort of fishing should be increased to reach maximum sustainable exploitation. Our findings would provide background data for future studies targeted at identifying factors that impact *L*_*m*_ and the spawning time, as well as defining the causes of fish mortality.

## 5 Conclusion

*Gagata cenia* is short and deep in body shape and reveals an imbalanced territory relative to the occurrence of higher predators in the Jamuna River. The maximum population stands on the 5.5–6.0 cm TL size group and *K*_*F*_ ascertained to be the distinguished parameter for estimating the well-being of this population in the Jamuna River. Since the *L*_*opt*_ and *L*_*m*_ are about the same in size, illicit gear should be disallowed, and mesh size should be raised to minimize capturing mature smaller individuals and allow them to spawn. Without it, the future stock will really be restricted owing to a shortage of spawners. Fishing mortality was significantly lower than natural mortality as well as exploitation rate signified under-exploited stock. This is the first effort to describe the biometric indices of *G. cenia* including growth pattern, growth parameters, reproduction, mortality, and exploitation rate from the Jamuna River as well from any water bodies and would be baseline study for the future studies.

## Declarations

### Author contribution statement

Zubyda Mushtari Nadia, Newton Saha & Prosun Roy: Conceived and designed the experiments; Performed the experiments; Analyzed and interpreted the data; Wrote the paper.

Md. Asif Iqbal: Performed the experiments; Wrote the paper.

Most. Shakila Sarmin & Md. Yeamin Hossain: Analyzed and interpreted the data; Contributed reagents, materials, analysis tools or data; Wrote the paper.

### Funding statement

This research did not receive any specific grant from funding agencies in the public, commercial, or not-for-profit sectors.

### Declaration of interests statement

The authors declare no conflict of interest.

### Additional information

No additional information is available for this paper.
